# Unveiling the Masked Culprit: Central Nervous System Infection With Varicella-Zoster Virus Diagnosed by Multiplex PCR in an Elderly Patient With Atypical Neurological Symptoms

**DOI:** 10.7759/cureus.71631

**Published:** 2024-10-16

**Authors:** Takafumi Uchi, Shingo Konno, Natsuki Inoue, Hideo Kihara, Hideki Sugimoto

**Affiliations:** 1 Department of Neurology, Toho University Ohashi Medical Center, Tokyo, JPN; 2 Department of Otolaryngology, Toho University Ohashi Medical Center, Tokyo, JPN

**Keywords:** central nervous system, dysphagia, hiccups, multiplex-pcr system, varicella-zoster virus

## Abstract

Varicella-zoster virus (VZV) infections of the central nervous system can be atypical, particularly in elderly patients. Herein, we describe the case of an 80-year-old male presenting with persistent hiccups and dysphagia without the characteristic rash typically associated with VZV infection. Cerebrospinal fluid multiplex polymerase chain reaction (PCR) allowed rapid identification of VZV, thereby enabling the prompt initiation of antiviral therapy. This case underscores the importance of considering VZV in atypical neurological presentations and highlights the utility of multiplex PCR in diagnosing zoster sine herpes, potentially altering the diagnostic approach for similar cases.

## Introduction

Varicella-zoster virus (VZV) infection of the central nervous system (CNS) causes a range of neurological manifestations. Although the classic presentation often includes a characteristic vesicular rash, atypical cases lacking cutaneous involvement, known as zoster sine herpes, can pose a significant diagnostic challenge.

Gilden et al. previously provided a comprehensive review of atypical presentations of VZV reactivation, highlighting their prevalence in elderly or immunocompromised patients [1]. In their extensive analysis, they emphasized that these reactivations can lead to diverse neurological symptoms, including the phenomenon known as zoster sine herpes, where a wide array of neurological complications can manifest without cutaneous involvement [1]. This work underscores the importance of considering VZV in the differential diagnosis of various neurological symptoms, even without the characteristic rash.

The advent of molecular diagnostic techniques, such as multiplex polymerase chain reaction (PCR), which offer superior sensitivity, specificity, and rapid turnaround times compared with conventional antibody tests, has revolutionized the approach to diagnosing CNS infections. Leber et al. demonstrated the efficacy of multiplex PCR (FilmArray® ME panel, bioMérieux SA Marcy-l'Étoile, France) in detecting various pathogens, including VZV, in the cerebrospinal fluid (CSF) [2]. However, its application to the atypical presentation of VZV infections has not yet been widely recognized.

Zoster sine herpes, first described by Lewis, remains a diagnostic challenge due to its nonspecific presentation [3]. Nagel and Gilden further elucidated the protean neurological manifestations of VZV infection, emphasizing the need for heightened clinical suspicion in cases lacking the typical rash [4].

This case report presents the case of an elderly patient with VZV CNS infection, manifesting primarily as persistent hiccups and dysphagia, without a typical rash. Herein, we highlight the role of CSF multiplex PCR in achieving a timely diagnosis, and discuss the implications for future diagnostic approaches in similar cases, building upon the work of Grahn and Studahl on VZV neurological complications [5].

## Case presentation

**Figure 1:**
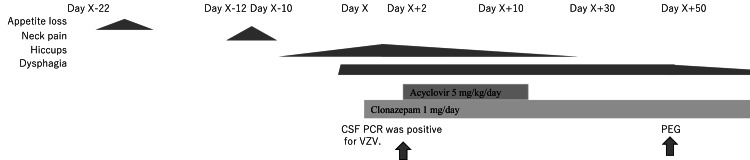
The clinical course of the case The patient initially presented with symptoms of appetite loss, neck pain, hiccups, and dysphagia, starting from Day X-22. These symptoms persisted and escalated over the next 10 days. On Day X+2, which was two days after admission, the diagnosis was confirmed with CSF PCR positive for VZV. Treatment was initiated with acyclovir at a dose of 5 mg/kg/day and clonazepam at 1 mg/day. The treatment continued, leading to significant improvement in symptoms by Day X+30. By Day X+50, a PEG was performed to provide nutritional support. CSF, cerebrospinal fluid; PCR, polymerase chain reaction; VZV, varicella-zoster virus; PEG, percutaneous endoscopic gastrostomy.

The patient’s clinical course is shown in Figure 1. 

The patient was an 80-year-old male who presented to our hospital with the chief complaint of dysphagia. His medical history revealed diabetes mellitus. He had been taking 1500 mg metformin and 100 mg sitagliptin phosphate hydrate per day for diabetes mellitus, 750 mg tranexamic acid, and 750 mg carbocisteine per day for pharyngeal dysphonia. The patient reported a progressive illness that began 22 days before admission (Day X-22), involving loss of appetite. Ten days later, he experienced neck pain, and two days after the onset of neck pain, frequent and prolonged hiccups lasting for hours developed. Subsequently, he developed dysphagia, which led to difficulty in oral intake.

**Figure 2:**
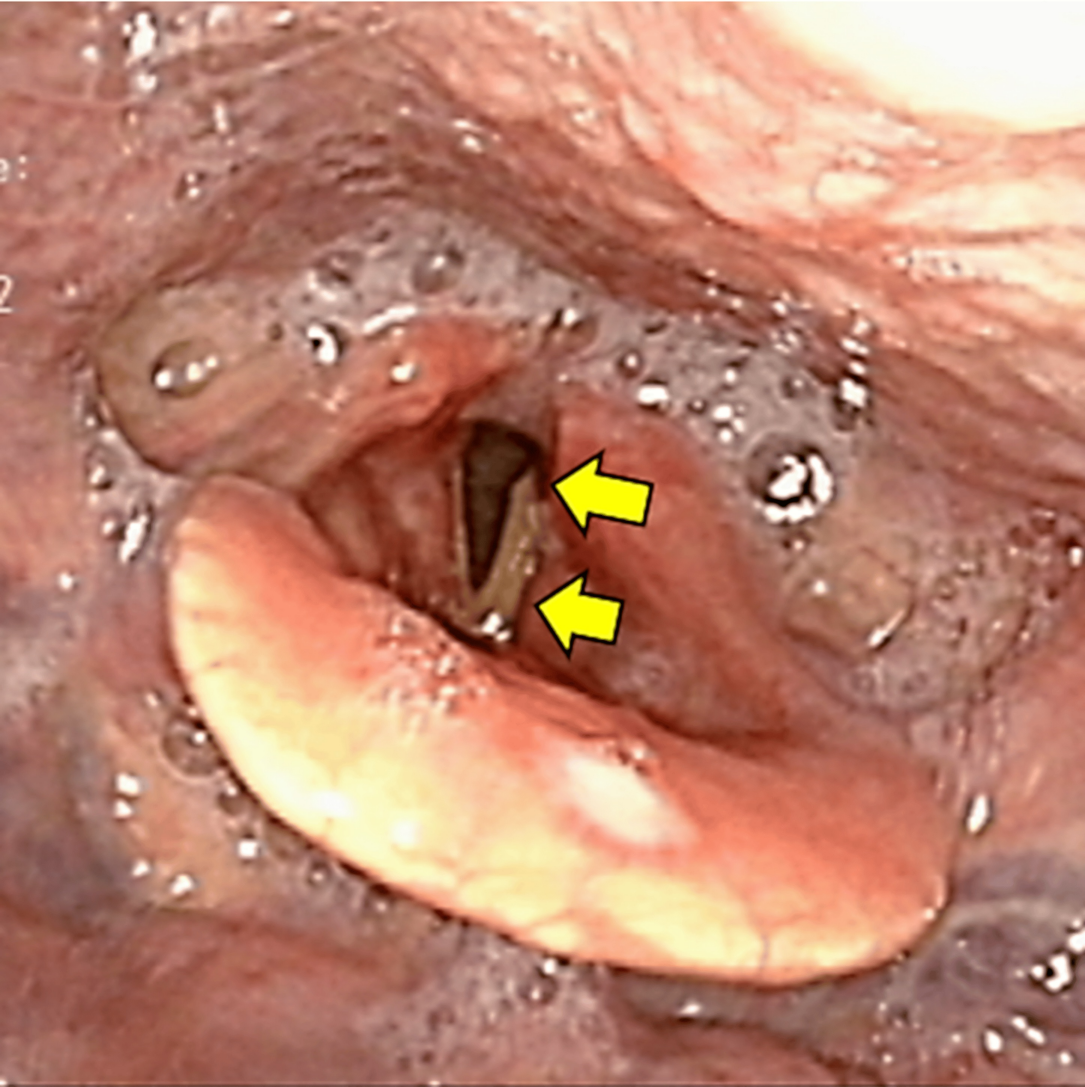
The swallowing endoscopy examination Laryngoscopy revealed several significant findings. First, there was an abundance of frothy saliva and laryngeal intrusion. The left vocal folds showed no movement and were less open than the right (yellow arrows). Additionally, the examination demonstrated decreased left pharyngeal contractions and delayed incomplete airway closure. These issues likely stem from impaired motor coordination and muscle control, possibly due to dysfunction of the nucleus ambiguus and vagus nerve. While these observations were evident in the laryngeal and pharyngeal regions, clear findings concerning the esophagus were difficult to observe.

On admission (Day X), General physical examination was unremarkable. Vital signs were within normal limits. The patient was alert and oriented. The skin was clear, and no lymphadenopathy was detected. Head and neck examination was normal. Lungs were clear to auscultation. A cardiovascular exam revealed a regular rate and rhythm without murmurs. The abdomen was soft and non-tender. Extremities showed no edema. Neurological examination further revealed no abnormalities in cranial nerve function, motor strength, or sensory perception, except for dysphagia. Notably, no involuntary movements were synchronized with the hiccups. The first swallowing endoscopy revealed prominent pharyngeal salivary effusion and left vocal cord paralysis (Figure 2).

Blood tests (Day X) and CSF tests (Day X+2) revealed the following abnormalities (Table 1).

**Table 1:** Abnormal findings in blood and cerebrospinal fluid tests Low sodium and low chloremia due to decreased food intake, increased blood glucose levels due to decreased fluid intake, and elevated anti-VZV antibody IgG in both blood and spinal fluid were observed. VZV, varicella-zoster virus; CSF, cerebrospinal fluid; IgG, immunoglobulin G.

Test	Measured Value	Normal Range
Serum		
Sodium (mEq/L)	123	136-145
Potassium (mEq/L)	3.8	3.5-5.1
Chloride (mEq/L)	86	98-107
Calcium (mg/dL)	9.4	8.5-10.2
Postprandial blood glucose (mg/dL)	319	70-140
HbA1c (%)	7.3	4.0-5.6
Anti-VZV-antibody IgG	11.2	<2.0
Anti-VZV-antibody IgM	0.39	<0.80
CSF		
Cell count (/mm³)	177	0-5
Polynuclear cells (/mm³)	170	0
Glucose (mg/dL)	120	40-70
Protein (mg/dL)	79	15-45
Anti-VZV-antibody IgG	>128	<2.0
Anti-VZV-antibody IgM	1.49	<0.80

**Figure 3:**
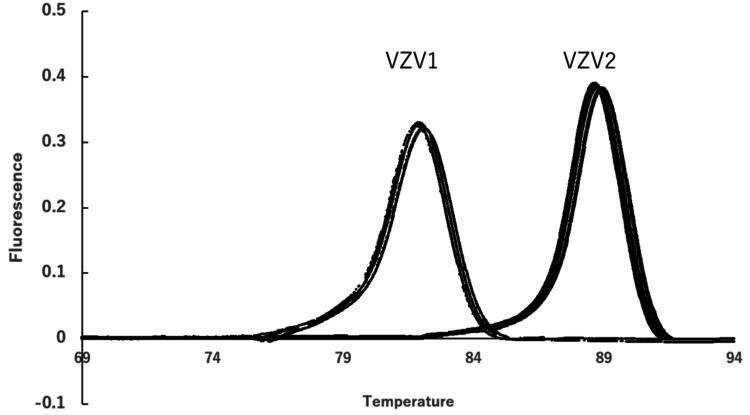
Melting curve analysis for detection of VZV targeting two distinct genetic regions (VZV1 and VZV2) Data acquisition and processing: The raw data for these analyses was acquired from a measurement device that does not support direct data export. As a result, the data was captured as a photograph of the device's display and then digitized using the free software available at https://automeris.io/. This process ensures that the plot accurately represents the original measurements, maintaining the integrity of the analysis. VZV, varicella-zoster virus.

Tests for anti-acetylcholine receptor antibodies and anti-muscle-specific kinase antibodies were negative. CSF analysis revealed a cell count of 177/mm³ (with 170/mm³ polynuclear cells), glucose 120 mg/dL, and protein 79 mg/dL. Multiplex-PCR testing of the CSF (FilmArray® ME panel, bioMérieux) was positive for VZV (Figure 3).

**Figure 4:**
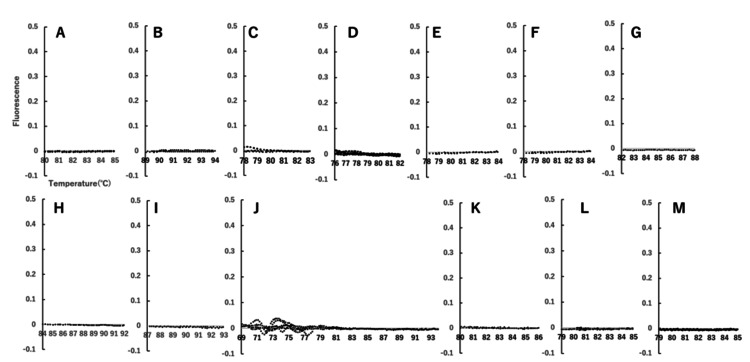
Melting curve analysis for detection of various pathogens in cerebrospinal fluid This figure illustrates the key result of our research - the confirmation of the absence of various pathogens in the tested sample. The x-axis represents the temperature range where melting peaks for the PCR products of each pathogen would be observed if present, while the y-axis shows fluorescence intensity. Each graph displays no significant melting peaks, providing strong evidence that the tested sample is negative for all pathogens analyzed. The pathogens include *Escherichia coli K1 *(A),* Haemophilus influenzae *(B),* Listeria monocytogenes *(C),* Neisseria meningitidis *(D),* Streptococcus agalactiae* (E), *Streptococcus pneumoniae* (F), cytomegalovirus (G), enterovirus (H), herpes simplex virus 1 (HSV-1) (I), HSV-2 (J), human herpesvirus 6 (K), human parechovirus (L), and *Cryptococcus neoformans*/*gattii* (M). The analysis includes an extended range on the x-axis for HSV2, reflecting the testing of two distinct genetic regions for this virus. Data acquisition and processing: The raw data for these analyses was acquired from a measurement device that does not support direct data export. Consequently, the data was captured as a photograph of the device's display and then digitized using the free software available at https://automeris.io/. This meticulous process ensures that the graphical representations accurately reflect the original measurements.

All other pathogens tested in the multiplex PCR panel were negative (Figure 4).

Bacterial and tuberculosis cultures of CSF were negative (culture result photographs are not available). The syphilis reaction in CSF was also negative.

**Figure 5:**
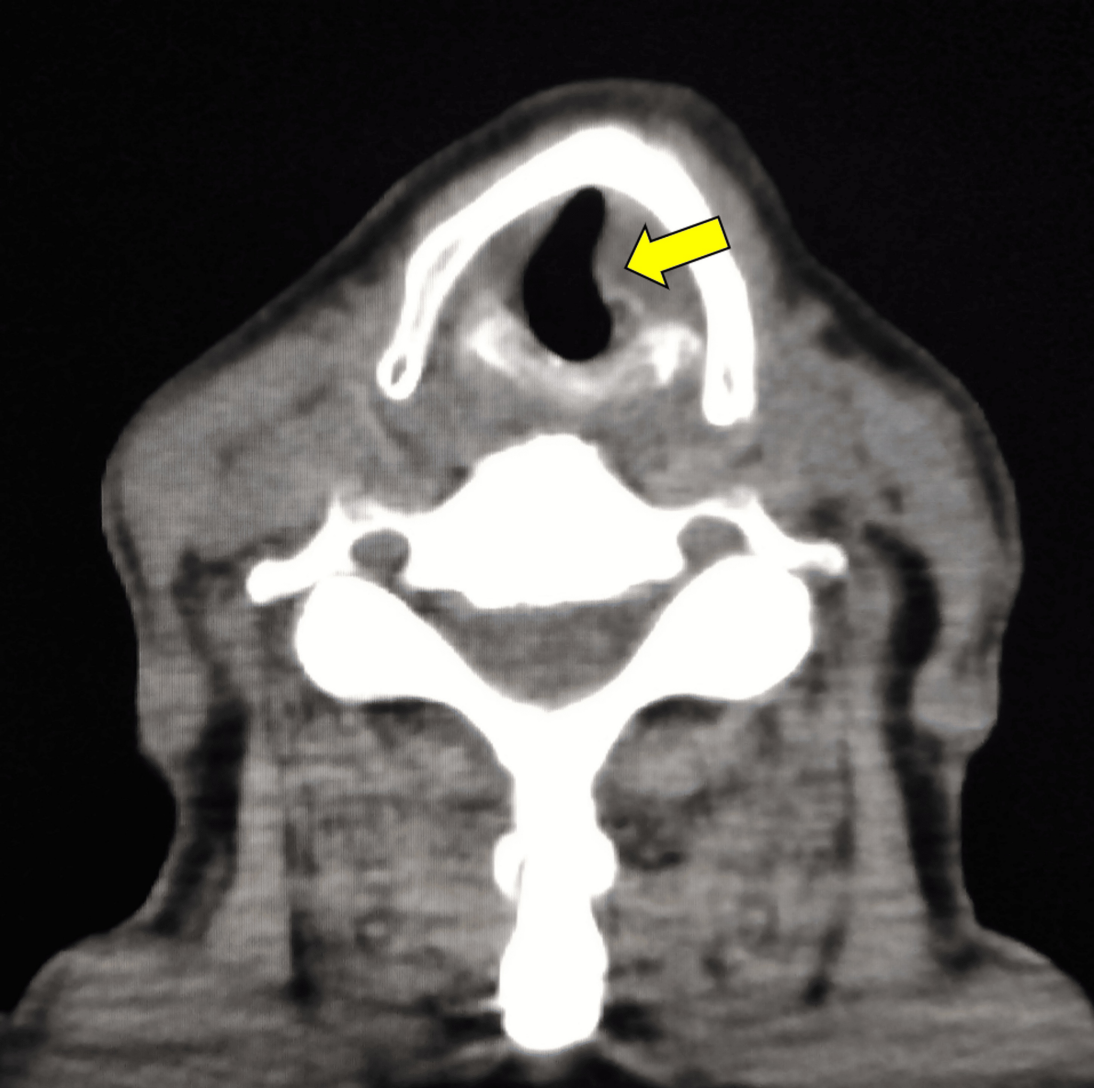
Computed tomography of the neck The picture of the slice on the vocal cord level showed an irregular shape of the edge in the left vocal cord compared to the right one (yellow arrow).

Brain magnetic resonance imaging (MRI) showed no abnormalities in the cerebrum, medulla oblongata, or visible cranial nerves with and without contrast enhancement. In contrast, cervical computed tomography (CT) revealed left vocal cord paralysis (Figure 5).

Based on multiplex-PCR results [2], intravenous acyclovir 5 mg/kg/day was started (Day X+2) and continued for 10 days (Day X+12). Clonazepam 1 mg/day was added to control hiccups (Day X). By day 30 of treatment, the patient showed significant improvement in hiccups. However, a second swallowing endoscopy revealed progressive disuse changes (Day X+14), indicating persistent dysphagia. The degree of left vocal cord paralysis remained unchanged; the second swallowing endoscopy also allowed observation of the esophagus, but no remarkable findings were noted. The hiccups improved, but the dysphagia persisted. Since long-term rehabilitation was necessary to restore swallowing, a PEG was performed on Day X+50, and the patient was transferred to a rehabilitation facility for continued care and swallowing therapy.

## Discussion

This case of VZV CNS infection presenting with hiccups and dysphagia without a typical rash exemplifies the concept of zoster sine herpes, a condition associated with a high risk of misdiagnosis or delayed treatment if not considered in the differential diagnosis. This is a diagnosis based on the following evidence. Generally, CSF of VZV-related CNS infection showed a mononuclear cell-dominant pattern. Interestingly, this case's CSF was polymorphonuclear cell-dominant pattern. The detection of many polymorphonuclear cells in the CSF of a VZV infection patient suggests an unusual inflammatory response, possibly due to the severe effects of the virus on the CNS and the resultant pro-inflammatory environment [6]. Neurosyphilis and tuberculosis were ruled out by CSF examination. Malignant lymphoma is difficult to differentiate by CSF examination but was considered negative based on the absence of abnormal CSF cytology and the lack of progressive course.

The neurological manifestations observed in this case, particularly persistent hiccups and dysphagia, are not among the most commonly reported symptoms of CNS VZV infections. However, Nagel and Gilden noted that VZV can affect various cranial nerves, leading to diverse neurological presentations [4]. This case adds to the literature on the atypical manifestations of VZV infections and underscores the importance of considering this diagnosis even in the absence of typical symptoms.

The rapid improvement in hiccups following acyclovir administration supports this diagnosis. It underscores the importance of early targeted therapy, as emphasized by Tunkel et al. in their guidelines for managing viral meningitis and encephalitis [7].

Despite improvements in other symptoms, the persistence of dysphagia in our patient highlights the potential for prolonged neurological deficits even after appropriate treatment. There have been reports of VZV infection with prolonged dysphagia similar to this case [8]. This phenomenon has also been observed in other cases of VZV CNS infections, as reported by Grahn and Studahl in their review of the neurological complications of VZV [5]. In our case, the need for long-term rehabilitation and PEG further emphasizes the potential severity and long-term impact of these infections. The natural course of VZV-related dysphagia without treatment is typically unfavorable, with a high likelihood of persistent symptoms and possible complications. Early diagnosis and treatment with antiviral medications like acyclovir are crucial to improve outcomes and prevent long-term sequelae [9].

In this case, it took three weeks to start treatment, resulting in a poor recovery of swallowing function. Therefore, no treated patient's functional recovery of swallowing should be feeble.

The successful use of CSF multiplex PCR for rapid diagnosis demonstrates its potential to significantly affect patient care, as indicated by Bahr and Boulware in their review of CNS infections in immunocompromised patients [10]. Integrating these advanced molecular diagnostic techniques into routine clinical practice could facilitate earlier diagnoses, more targeted treatments, and potentially improved outcomes in similar cases.

A significant limitation of this case report is its inherent nature as a single-patient study, which constrains the generalizability of our findings to a broader patient population. The observed outcomes may be influenced by unique patient characteristics or circumstances that are not representative of other cases, further limiting the applicability of our conclusions. Additionally, the absence of long-term follow-up data restricts our ability to assess the durability of the observed effects over time. It is important to note that, due to the nature of a case report, we cannot establish a causal relationship between the intervention and the outcome with certainty. Furthermore, the possibility of reporting bias should be considered, as positive outcomes are generally more likely to be reported in the literature than negative ones.

Future research should focus on elucidating the full spectrum of neurological manifestations associated with VZV CNS infections and optimizing the diagnostic and treatment strategies for atypical presentations, particularly among elderly and immunocompromised populations. Additionally, studies on the long-term outcomes and rehabilitation strategies for patients with persistent neurological deficits following VZV CNS infections are warranted.

## Conclusions

This case report contributes to the growing body of evidence supporting the use of multiplex PCR to diagnose CNS infections, particularly herpes zoster. This case further emphasizes the need for clinicians to maintain a high index of suspicion for VZV infection in elderly patients presenting with unexplained neurological symptoms, even in the absence of typical dermatological findings. This case highlights the importance of considering the atypical presentation of VZV infections and the potential for rapid diagnosis using advanced molecular techniques that can lead to timely treatment and improved patient outcomes.
